# Tramadol for the Management of Opioid Withdrawal: A Systematic Review of Randomized Clinical Trials

**DOI:** 10.7759/cureus.9128

**Published:** 2020-07-11

**Authors:** Kaushal Shah, Billy Stout, Hunter Caskey

**Affiliations:** 1 Psychiatry, Griffin Memorial Hospital, Norman, USA; 2 Psychiatry and Behavioral Sciences, The Recovery Center, Oklahoma City, USA; 3 Psychiatry and Behavioral Sciences, Griffin Memorial Hospital, Norman, USA

**Keywords:** opioid medication, opioid reversal, opioid withdrawal, detox, ows, methadone, buprenorphine, cows, cgi, dependence

## Abstract

The increase in the prescription of opioid medications has resulted in a wildfire of misuse of opioids, both for medical and non-medical reasons, with over 1.7 million people in the United States (US) suffering from distinct disorders owing to opioid use. While various medications, such as methadone, buprenorphine, and naloxone, among others, have been used in treating opioid withdrawal symptoms, concerns of the potential abuse of these drugs, the cost of procurement, legislations, and prescription policies have risen. In recent times, tramadol has been considered a viable replacement for some of these treatment regimes. Tramadol is a synthetic analgesic that acts centrally, possessing opioid-like effects due to the binding of its metabolite with the mu (µ)-opioid receptor, yet with low potential for abuse. Several clinical studies conducted in the past ten years have identified the effects of tramadol in opioid withdrawal cases. The results showed that it exhibits better efficacy and tolerance with fewer side effects in specific clinical scenarios as compared to existing available detox management. We aim to examine the properties of tramadol in opioid withdrawal through this systematic review of clinical studies on humans.

## Introduction and background

The use of opioids dates back to more than 5000 years, occurring as natural extracts of the poppy plant, 'Papaver somniferum,' and it can also be synthesized [1-2]. Since the early 20th century, opioids have been accessed easily in the United States (US) and used for headaches, toothache, diarrhea, insomnia, anxiety, and cough, among others, and it has resulted in widespread opioid abuse and dependence [3]. The feelings of euphoria, tranquility, and sedation produced by opioids have led individuals to continue seeking the drug, especially in doses that overwhelm the respiratory drive and lead to death [4].

The Diagnostic and Statistical Manual of Mental Health Disorders (DSM-5) published by the American Psychiatric Association (APA) in 2013 defined opioid use disorder (OUD), as the repeated occurrence within 12 months of two or more of 11 problems, including withdrawal, giving up life events to use opioids, and excessive time spent using opioids [5]. A 2010 global epidemiology and burden of opioid dependence study showed that there were 15.5 million opioid-dependent people globally, accounting for 9.2 million disability-adjusted life years (DALYs) [6]. While some 12-million Americans are estimated to misuse opioids and about two million diagnosed with OUD, with an average of five deaths per hour due to opioid overdose, it costs the US economy 504 billion dollars annually [7-8]. Three-million eight-hundred thousand people in the United States aged 12 and older reported past-month misuse of prescription pain medication in 2015 [9]. Opioid use disorder prevalence among pregnant women increased from one and a half to six and a half cases per 1000 delivery hospitalizations from 1999-2014 [10]. Approximately 500,000 people died from opioid overdoses from 2000 to 2015 [11].

As a response to the opioid epidemic, in March 2015, the Centres for Disease Control and Prevention (CDC) released the 'Guideline for Prescribing Opioids for Chronic Pain,' stipulating that a quantity not higher than needed for the expected duration of pain severe enough to require opioids be given [12]. And as of 2018, about 33 states had enacted legislation ordering on opioids prescription limits [12]. Drugs such as methadone, buprenorphine, naloxone, and clonidine have been used in the last three decades for treating opioid withdrawal [13]. Methadone is used for both withdrawal and maintenance treatment but is limited by regulations that make it available only through licensed narcotic treatment centers [14]. Treatment with buprenorphine, while useful, is limited by its prolonged half-life, making it challenging to switch medication, and has a potential for abuse [15]. Complications of symptomatic hypotension and bradycardia are some of the limitations of the use of clonidine in opioid withdrawal, leading to a low rate of treatment-seeking and a high detoxification failure rate [14].

Given the above, recent studies have sought alternatives for the treatment of opioid withdrawal. Tramadol has shown to exhibit opiate receptor activity. With its low potential for abuse, it is considered to have potential as an effective treatment for opioid withdrawal [16]. This systematic review aims to assess the effects of tramadol in opioid withdrawal management through qualitative analysis of past randomized controlled trials on humans.

## Review

Study methodology

**Figure 1:**
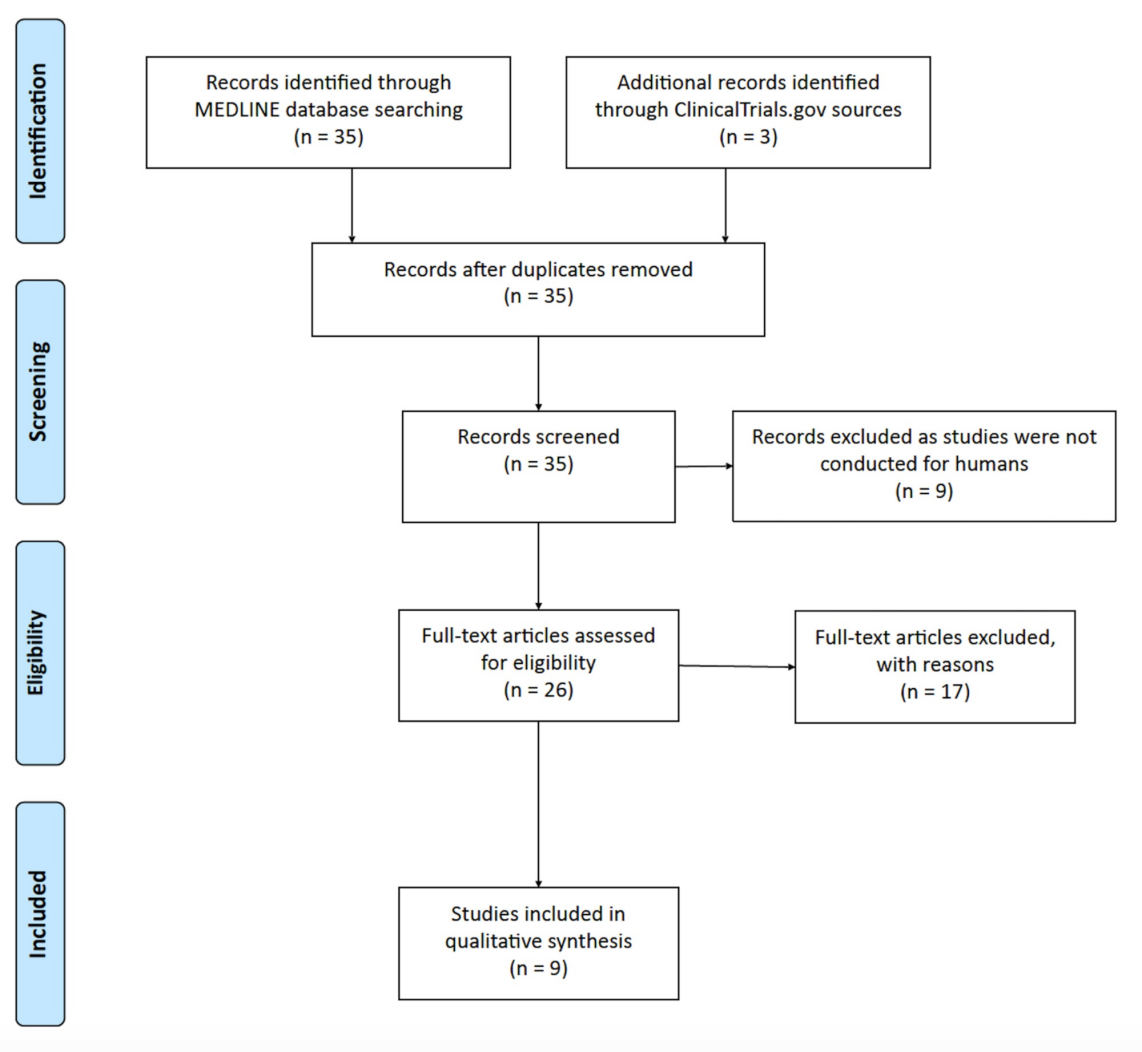
Results of the systematic review according to Preferred Reporting Items for Systematic Reviews and Meta-Analysis (PRISMA) guidelines

We searched the Medline database of the National Library of Medicine (NLM) for clinical studies between January 1, 2000, and June 15, 2020. A similar strategy was adapted to search the ClinicalTrials.gov database for registered clinical trials. Clinical trials in all phases, comparative studies, and clinical studies were examined by using the search term, 'Tramadol' in the context of 'Opioid Withdrawal' and 'Opioid Detox,' and that generated 35 studies in the results. In accordance with the Preferred Reporting Items for Systematic Reviews and Meta-Analyses (PRISMA) guidelines, all searches and screening of the studies were performed by the two independent authors (K Shah and H Caskey). After reviewing study titles and abstracts, we excluded nine non-human studies and three duplicate studies. A further detailed review of full-texts of the studies determined nine eligible clinical studies for the qualitative synthesis of our research, as shown in Figure 1.

Overview of tramadol

Tramadol is a 2-(dimethylamino)-methyl-1-(3-methoxyphenyl) cyclohexanol hydrochloride and a 4-phenyl-piperidine analog of opioid drug codeine, which was discovered and synthesized in 1962 by a German company for pain treatment. It was introduced by the name tramadol into the market in 1977 and became available in the US market in 1995 [17].

Prescriptions for tramadol have seen a steady rise from 23.3 million in 2008 to 44 million in 2013 in the US alone. While it is considered safe owing to the lower risk of tolerance, abuse, and dependence, it is said to have less clinical value than other opiates, with only about one-tenth the pain-reducing qualities of morphine [17].

Tramadol is primarily used for the treatment of muscle, joint, and wound pain and is usually not recommended for those under 16 years of age and those with a medical history of kidney disease, liver disease, stomach disorder, mental illness, depression, and suicidal ideation [18]. The Drug Enforcement Administration (DEA) in 2014 reported that about 3.2 million people above the age of 12 in the US took tramadol for non-medical reasons [17].

Opioid withdrawal, dependence, and maintenance

Opioid withdrawal symptoms (OWS) have different levels of severity and duration, depending on the half-life of the opioid, length of usage, and patient's individual characteristics such as health status. The most common symptoms include aches, muscle spasms, tremor, abdominal cramps, nausea, vomiting, anxiety, irritability, insomnia, hot flashes, heart-pounding, lacrimation, and sweating [1].

Sudden stoppage of short-acting opioids (e.g., heroin) results in severe OWS, which usually begins within 12 hours following a dosage miss and can last four to seven days while withdrawal for long-acting opioids (e.g., buprenorphine) could be less severe under similar duration [19]. It has been noted that the avoidance of OWS becomes the most compelling reason for continued usage, despite the pain relief, relaxation, self-medication, or pleasure-seeking, which may have informed a primary reason [20]. The physical dependence on opioids can be developed within days to weeks following repeated daily usage; tolerance and complete dependence become inevitable with prolonged usage [1].

Opioid maintenance therapy, also known as agonist replacement or agonist assisted therapy, is the administration of a prescribed opioid substance usually under medical supervision to prevent the emergence of withdrawal symptoms and reduce cravings. Methadone offered orally and buprenorphine given sublingually are often used for opioid maintenance therapy [21].

Drugs for the opioid withdrawal treatment

The American Society of Addiction Medicine-approved medications for opioid addiction treatment are listed in Table 1 [21].

**Table 1:** Medications for opioid addiction treatment

Medications	Indications
Methadone	Opioid use disorder, opioid withdrawal, and opioid maintenance management
Buprenorphine	Opioid use disorder and opioid withdrawal management
Clonidine	Opioid use disorder and opioid withdrawal management
Naltrexone	Opioid relapse prevention
Naloxone	Opioid overdose

Methadone is a slow-acting opioid agonist that has been considered useful in opioid withdrawal management. It is taken orally to dampen the euphoria associated with using other administration routes, thereby preventing OWS. Several studies have shown it to be preferred above abstinence-based approaches [22]. Treatment begins with an initial dose range from 10 mg to 30 mg, depending on the level of physical dependence evaluated in the first two to four hours. Acute withdrawal symptoms are often reduced by relatively low doses of less than 30 mg per day, but such doses may not be able to suppress craving. While some patients respond to a maintenance dose of 30 mg to 60 mg per day, most do better with a steady and gradual increase in treatment dose of 60 mg to 120 mg. However, doses of 80 mg to 100 mg per day have been shown to produce better outcomes in randomized trials [23]. Cases of intolerable methadone side effects, lack of successful treatment outcomes, or a patient's desire to change treatment and suitability for an alternative regimen can result in switching to other opioid treatment medications [21].

Buprenorphine has shown to be an effective treatment for OWS, with a lower risk of lethal overdose in opioid-tolerant individuals as compared to other opioid medications [24]. It is unique for inhibiting drug cravings void of the euphoric side effects associated with other opioids [21]. Initial doses of 2 mg to 4 mg are recommended; later, patients are assessed within 60 minutes to 90 minutes for signs of precipitated withdrawal and in their absence, the dose is increased by 2 mg to 4 mg. Many patients tolerate the drug well, but in cases of adverse side effects or no successful course of treatment, a switch can occur [24].

Naltrexone, a long-acting opioid antagonist with the properties of blocking the effects of opioids, is used to help relapse [21]. A review of 13 randomized controlled clinical trials, with a total of 1158 participants, compared treatment with oral naltrexone against either placebo or no medication for OUD and showed that oral naltrexone is not superior in relapse prevention, but more effective in sustaining abstinence [25]. According to the guidelines provided by the American Society of Addiction Medicine (ASAM), the recommended dosage of oral naltrexone is 50 mg per day or two 100 mg doses three times a week, followed by one 150 mg dose. The injectable naltrexone can be administered intramuscularly every four weeks [21]. A clinical trial evaluation of a once-per-month extended-release injectable naltrexone formulation for patients with difficulty adhering to daily medication found effectiveness in treating polysubstance dependency with the reduced use of heroin and amphetamines [26].

An alpha-2 adrenergic agonist such as clonidine is an alternative non-opioid treatment method for withdrawal treatment, usually dosed at 0.1 mg to 0.3 mg every six to eight hours with a maximum dose of 1.2 mg [21].

Pharmacology and mechanism of action of tramadol

Tramadol is a centrally acting analgesic that acts by changing the ascending pain pathways through partial agonism of mu (µ)-opioid receptors and serotonin and norepinephrine inhibitors, undergoing an extensive metabolism hepatically and forming an active metabolite via CYP2D6 [27]. It possesses two chiral centers and is used as a 1:1 racemic mixture of 2 enantiomeric diastereomers, the R, R-enantiomer ((+) - tramadol) and S, S-enantiomer ((-) -tramadol). The (+)-tramadol enantiomer is the most potent serotonin reuptake inhibitor while the (-)- tramadol enantiomer is the most potent norepinephrine and serotonin reuptake inhibitor [28].

Tramadol is metabolized in two phases, with phase I reactions being slower than phase II. The phase I is catalyzed by cytochrome P450 2D6 (CYP2D6) and cytochrome P450 3A4 (CYP3A4), with the Ο-demethylation reaction to the active O-desmethyl tramadol (M1) metabolite catalyzed by CYP2D6 [29]. Metabolism of tramadol occurs in the liver, and the unchanged tramadol and its metabolites are excreted in urine [30]. It exhibits some opioid agonist-like effect, with a lower potential for abuse as compared to typical analgesics [31]. In a prospective human laboratory study, oral tramadol doses of 200 mg to 400 mg were shown to bring about moderate opioid withdrawal suppression [32].

Efficacy of tramadol in the management of opioid withdrawal

In this study, nine randomized control trials were reviewed for the qualitative analysis of the evidence of tramadol's effectiveness and efficacy in opioid withdrawal.

Randomized Controlled Trial # 1

In another double-blinded, randomized control trial (RCT), 10 residential opioid-dependent adults participated to understand the suppression efficacy of oral tramadol for opioid withdrawal. Opioid-dependent adults were maintained on morphine for a six-week duration and induced withdrawal before the experimental sessions to assess the effect of placebo, tramadol (50 mg, 100 mg, 200 mg, and 400 mg orally), naloxone (0.1 mg and 0.2 mg intramuscular), and morphine (15 mg and 30 mg intramuscular) [33].

Morphine and naloxone were observed to produce a dose-related increase of typical opioid agonist and antagonist effects, respectively, while the effects of tramadol varied per individual and depended on time and dose. Effects of 50 mg and 100 mg tramadol were similar to placebo, while 200 mg and 400 mg yielded evidence of suppression of opioid withdrawal. However, that suppression was slower than seen with morphine, which could be a result of differences in routes of administration as well as the conversion of tramadol to its active metabolite, M1. The researchers noted that tramadol is not linked with significant positive drug effects such as feeling high, drug liking, or good effects, suggesting that acute doses of tramadol show low abuse liability when used in opioid withdrawal. It also provided evidence of its effectiveness in opioid withdrawal with 200 mg and 400 mg doses without subject and observer-rated agonist effect [33].

Randomized Controlled Trial # 2

A total of 60 patients with heroin dependence were recruited to compare the effectiveness of tramadol with clonidine in opioid withdrawal. An equal number of participants received clonidine treatment and tramadol treatment. The clonidine group was administered 300 mg to 500 mg given three to four times per day, while the tramadol group had 200 mg to 300 mg with two to three doses per day. The clinical opioid withdrawal scale (COWS) was administered in all participants to evaluate the efficacy of both treatment groups. Results showed that treatment with tramadol reduced withdrawal symptoms in all 11 items on COWS, and mean withdrawal scores were lower on subsequent days for the tramadol group than the clonidine group. As tramadol was more effective in preventing sweating, restlessness, aches, runny nose, gastrointestinal (GI) upset, yawning, anxiety, and goose skin, the study concluded that it could be effectively used in opioid withdrawal in outpatient treatment settings [34].

Randomized Controlled Trial # 3

Lanier et al. assessed the opioid blockage efficacy and level of physical dependence in four weeks of randomized, double-blind, cross-over-design clinical trials [35]. Nine residential opioid-dependent adults maintained on two doses daily of oral tramadol (200 mg and 800 mg) to test the acute effects of intramuscular placebo, naloxone (0.25 mg, 0.5 mg, and 1.0 mg), and hydromorphone (1.5 mg, 3.0 mg, and 6 mg), in a series of seven experimental sessions done 48 hours apart. Findings showed that the challenge dose of 0.5 mg and 1.0 mg naloxone in the course of 800 mg per day tramadol dose produced an elevation in participant- and observer-rated measures of antagonist effects, which were similar in magnitude to scores obtained with 0.5 mg naloxone in maintenance with 60 mg per day subcutaneous morphine. Withdrawal severity from naloxone was seen to be greater with 800 mg versus 200 mg per day tramadol [35].

Findings suggest that tramadol may exhibit low opioid-like subjective effects in human beings, which makes it have low abuse potential. It was also found to create dose-related opioid physical dependence but does not produce dose-related attenuation of agonist challenge effects. No evidence was found for opioid cross-tolerance linkage to daily tramadol administration. Hence, it may be useful in treating individuals with opioid withdrawal or as a detoxification agent but not for maintenance treatment due to the risk of physical dependence owing to prolonged use [35-36].

Randomized Controlled Trial # 4

In a study by Duke et al., the discriminative stimulus effects of tramadol in humans were assessed [37]. Eight non-dependent volunteers with active stimulant and opioid use were trained to discriminate between placebo, hydromorphone (8 mg), and methylphenidate (60 mg). Participants were given samples of each and informed that they might either experience no effects, stimulant effects, or other effects and were to pay attention to the effects of each letter-coded drug. The participants underwent three phases: discrimination training to identify each condition by letter code, a test to assess if each participant could correctly identify each drug condition by letter code, and discrimination test sessions. Doses of hydromorphone (4 mg and 8 mg), methylphenidate (30 mg and 60 mg), tramadol (50 mg, 100 mg, 200 mg, and 400 mg), and placebo were tested. Through the visual analog scale (VAS), participants rated drug effects as high, like good effects, bad effects, sick, desire for cocaine now, similar to opioids, and similar to stimulants [37].

Results showed that participants were able to identify each drug condition at least once correctly. Doses of hydromorphone were majorly identified as an opioid agonist, doses of methylphenidate as a stimulant, while lower doses of tramadol were identified as placebo and higher doses as an opioid agonist. Hydromorphone (8 mg) was observed to increase VAS ratings of like and good effects compared to placebo, hydromorphone, and methylphenidate's increased ratings of high and drug effect; tramadol did not significantly increase ratings of like or good effects [37].

Overall, tramadol did not increase ratings of drug liking, good effects, high or drug effects significantly, though it majorly increased scores on the stimulant scale at higher doses, which could be attributed to its slow onset and lower efficacy at µ-opioid receptors in comparison with full µ-opioid agonists [35-36]. The researchers noted that these findings suggest tramadol as a useful treatment option for low-level opioid dependence or mild to moderate opioid withdrawal and along the lines with the findings of Lanier and Threlkeld [35,38].

Randomized Controlled Trial # 5

Seventy participants took part in a double-blind, randomized clinical study to compare the efficacy and safety of tramadol and methadone for opioid withdrawal treatment. Participants randomized to either 600 mg of tramadol (200 mg three times a day) or 60 mg of methadone (20 mg three times a day), with a 20% tapered dose subsequently every two days for 11 days [39].

Analysis of results did not show a significant difference in OOWS scores of the two treatments, though pain rates observed in the methadone group were five times higher than the tramadol group. Even though the retention rate was similar in both the groups, but the side effects were less in the tramadol group. The researchers concluded that the tramadol could be a safe substitute to low or medium doses of methadone for rapid heroin detoxification. Zarghami et al. concluded that the tramadol should be considered a potential substitute for methadone due to similar efficacy but found less effective than medium doses of methadone to control withdrawal symptoms [39].

Randomized Controlled Trial # 6

A two-phased RCT trial to assess the effects of extended-release (ER) tramadol for the treatment of prescription opioid withdrawal, was conducted, involving participants aged 18-55 with at least 21 days of non-medical use of a short-acting prescription opioid in the last 30 days and consented to be part of an inpatient opioid detoxification for 14 days. During the first phase (day one to seven), participants were grouped into three using a double-blind, placebo-controlled parallel-group design. They were administered a daily oral placebo twice or ER tramadol (200 mg or 600 mg daily, given in two divided doses). While in the second phase (Day 8 to 13), those that were on tramadol phase 1 had a double-blind cross over to placebo, and those initially on placebo in Phase 1 stayed on. Primary outcome measures were the number of breakthrough withdrawal doses received and total subject-rated opioid withdrawal adjective score [40].

From the findings, there were significant differences between the groups on the amount of breakthrough withdrawal medication administered, with the 200 mg tramadol group in Phase 1 requiring the least quantity of breakthrough doses than placebo and 600 mg tramadol group. It also exhibited lower peak ratings on different withdrawal items, e.g., insomnia. Those transferred to placebo from 600 mg tramadol during phase 2 were given the most breakthrough medication, with significantly higher total ratings of opioid withdrawal on two observer-rated measures and higher peak ratings on several withdrawal signs. The doses used in the study were well-tolerated, safe, and did not show any signs of abuse liability [40].

The researchers noted that while the results provide a positive signal of clinical efficacy, there was no clarity as to whether the differences observed were substantial enough to be clinically effective. However, they agreed that findings suggest that optimal repeated dosing of tramadol for the treatment of opioid withdrawal may be within the maximum analgesic range dose of ER 300 mg or less than 600 mg if above it required [40].

Randomized Controlled Trial # 7

Comparison study conducted by Chawla et al. to assess the efficacy between buprenorphine and tramadol in the detoxification of opioid (heroin) dependent subjects, involving 62 subjects aged 20-45 years, showed that mean scores on SOWS, objective opiate withdrawal scale (OOWS) and VAS list, were significantly lower for the buprenorphine group on the second and third day than for the tramadol group. Tramadol was observed to have limited efficacy in treating moderate to severe opioid withdrawal compared to buprenorphine [41].

Randomized Controlled Trial # 8

A comparative study of tramadol and buprenorphine for the treatment of opioid withdrawal conducted by Pahwa et al. that had 70 participants with heroin as the drug of choice with opioid physical dependence. They were grouped for mild (<10 mg), moderate (10 mg to 20 mg), and severe (>20 mg) heroin drug use and assigned into 2 mg buprenorphine and 100 mg tramadol. Objective and subjective evaluation was done based on clinical opioid withdrawal scale (COWS) and clinical global impression (CGI) [42].

In the mild user group, results showed that 53.33% had early full remission, and 33.33% had early partial remission, while those in the buprenorphine group had 36% and 46.6%, respectively. The moderate group witnessed 35.71% full remission and 42.85% partial remission for tramadol, while 21.42% had full remission and 53.17% partial remission for buprenorphine. Patients treated with tramadol had higher average withdrawal symptoms and a greater reduction in withdrawal symptoms over time than buprenorphine. A higher number of moderate users of heroin completed detoxification treatment in the tramadol group than buprenorphine (78% vs. 57%). However, tramadol had high drop-out rates amongst patients with a severe level of addiction. The study showed that tramadol has better efficacy in relapse prevention and detoxification amongst the moderate level of opioid-dependent users as compared to buprenorphine [42].

Randomized Controlled Trial # 9

Dunn et al. studied the efficacy of extended-release (ER) tramadol for opioid withdrawal and recruited 103 participants aged 18 to 60 years, who met DSM-IV criteria for opioid dependence [43]. The study was divided into three phases, phase 1, which involved treatment with morphine 30 mg, a naloxone challenge, and subsequent randomization into groups to receive clonidine, tramadol ER, and buprenorphine. In phase 2, medications were tapered in a double-blind, double-dummy fashion, with participants receiving one capsule orally, which contained either clonidine, tramadol or placebo, and four 2 mg-sized sublingual tablets of either buprenorphine hydrochloride or placebo, once daily. Phase 3 witnessed a cross -over of participants to placebo capsules tablets. Participants were made to complete the subjective opioid withdrawal scale (SOWS) [43].

From the results, tramadol was observed to produce retention and greater withdrawal suppression than clonidine and comparable to buprenorphine. SOWS withdrawal ratings decreased among clonidine and tramadol ER participants, while buprenorphine participants had a slight increase. Treatment with tramadol ER did not yield an increase in withdrawal symptoms following the medication taper, supporting its use for opioid withdrawal treatment [43].

Our findings of the systematic review of clinical studies are shown in Table 2 [33-35,37,39-43].

**Table 2:** A synopsis of the clinical studies of tramadol for the treatment of opioid withdrawal

Source	Study purpose	Conclusion
Lofwall et al., 2007 [33]	The purpose was to characterize the opioid withdrawal suppression efficacy of oral tramadol.	Tramadol showed evidence of opioid withdrawal suppression without significant observer and subject-rated opioid agonist effects. It did not show a high risk of tramadol abuse in opioid-dependent individuals. Tramadol is a useful medication for treating opioid withdrawal.
Chattopadhyay et al., 2010 [34]	The study compared the effectiveness of tramadol with clonidine in opioid withdrawal.	Tramadol found to be more effective in preventing sweating, restlessness, aches, runny nose, gastrointestinal upset, yawning, anxiety, and goose skin. It can be used effectively in the opioid withdrawal in outpatient treatment settings.
Lanier et al., 2010 [35]	The study assessed the level of physical dependence and opioid blockade efficacy produced by daily maintenance on oral tramadol.	It found tramadol a useful treatment for patients with low levels of opioid dependence or as a treatment for withdrawal during opioid detoxification, but it is not effective as a maintenance medication due to a lack of opioid cross-tolerance.
Duke et al., 2011 [37]	Assessed the discriminative stimulus effects of tramadol in humans.	Tramadol did increase subjective ratings associated with reinforcement. It is a potential medication for the treatment of opioid dependence and withdrawal.
Zarghami et al., 2012 [39]	The study compared the efficacy and safety of tramadol versus methadone for the treatment of opiate withdrawal.	Tramadol could be considered as a potential substitute for methadone to manage opioids withdrawal and may be as effective as methadone.
Lofwall et al., 2013 [40]	Evaluated efficacy of extended-release tramadol in treating opioid withdrawal.	A lower dose of extended-release tramadol modestly attenuates opioid withdrawal. It is a possible treatment of opioid withdrawal.
Chawla et al., 2013 [41]	The study compared tramadol and buprenorphine for controlling withdrawal symptoms in patients with opioid dependence syndrome.	Tramadol showed limited detoxification efficacy in moderate to severe opioid withdrawal as compared to buprenorphine. It has potential use in mild to moderate opioid withdrawal cases.
Pahwa et al., 2015 [42]	It compared the effects of tramadol and buprenorphine in the treatment of heroin withdrawal in opioid dependant patients.	Tramadol is not superior to buprenorphine in opioid maintenance treatment. It showed effectiveness in detoxification and relapse prevention in patients with a moderate level of opioid dependence and withdrawal as compared to buprenorphine.
Dunn et al., 2017 [43]	Evaluated the effectiveness of extended-release tramadol in the treatment of opioid withdrawal.	Study results suggested that tramadol extended-release is more effective than clonidine and comparable to buprenorphine in reducing opioid withdrawal symptoms during a residential tapering program.

Limitations

While the researchers used similar or different approaches in their clinical studies, it is vital to be aware of these limiting factors: a) Failure to draw blood for the pharmacokinetic evaluation of tramadol [35]; b) Impact of small sample sizes of the trials on the statistical significance [34-35,42]; c) Participants in outpatient settings were akin to exhibiting non-compliance [34]; d) The study participants' data regarding the assessment of heroin use and dependence may have been affected by the recall or reporting bias [39]; e) The possibility of concurrent abuse may have affected the nature and severity of withdrawal symptoms or side effects of the treatment regime [39]; f) Some of the studies excluded persons dependent primarily on heroin [33,37].

Comparison of tramadol with other treatments

Several studies have shown that tramadol could be effective in an opioid detox [33]. While other treatment regimes exist, it is crucial to review tramadol's efficacy in comparison with some of these drugs.

A study by Zarghami et al. discovered that there was no significant difference between the COWS scores in the treatment of 70 patients with methadone and tramadol; also, the side effect scores were not significantly different [39]. However, they noted that the rates of pain were five times higher in methadone than in tramadol. Furthermore, they stated that tramadol has a low abuse potential, better pain-reducing efficacy, and a short half-life, making it more preferred than methadone. Likewise, Salehi et al. suggested that tramadol is more effective, especially when used for detoxification in outpatient settings [44].

Effective treatment of opioid withdrawal with buprenorphine has been established, and it has the ability to inhibit drug cravings and, with a lower risk of lethal overdose, makes it highly recommended [24,45-46]. However, Chawla et al., 2013, in their study, determined that buprenorphine has a higher potential for abuse than tramadol [41]. They also determined that tramadol was efficient in detoxification and relapse prevention in patients with a moderate level of dependence, while buprenorphine is better for maintenance treatment and in severe dependence [41,44].

Tramadol was found more effective in opioid detox than clonidine, reducing all symptoms on COWS [36]. Likewise, a study of 59 participants detoxified with tramadol and 85 detoxified with clonidine observed that those treated with tramadol were less likely to leave against medical advice as compared to those with clonidine, which was suggestive of it being more effective than clonidine [45]. Duke et al. in their study found that hydromorphone increased VAS ratings of like and good effects, and together with methylphenidate, ratings of high and drug effects were increased as compared to that of placebo. Tramadol did not significantly increase such effects, gaining an edge over them [37].

In the course of the respective RCTs, researchers noted that most of the participants exhibited good tolerance for tramadol use. However, a participant on tramadol 200 mg complaining of left-sided flank pain was recorded [33]. Though the symptoms resolved without any intervention, some complained of aches in their teeth, an onset of a non-pruritic painful rash, which was treated with topical hydrocortisone. Participants also reported cases of vomiting after ingestion of 200 mg and after 400 mg tramadol, stomach-turning [33,35]. Some of the studies did not differentiate adverse events from opioid withdrawal symptoms [43].

## Conclusions

There is sufficient evidence showing the efficacy of tramadol in treating opioid withdrawal, considering that it has a low liability for abuse and has good tolerance. Tramadol has demonstrated valuable potential in managing mild to moderate withdrawal cases at higher doses than prescribed for analgesia. It has also shown promises as a treatment option in an outpatient setting. Further well-designed studies and clinical trials are imperative with the inclusion of a broader array of variables to ascertain its comprehensive role in immediate and long-term care for opioid withdrawal management, including dosing, safety, efficacy, and contraindications.
